# Complexation with C_60_ Fullerene Increases Doxorubicin Efficiency against Leukemic Cells In Vitro

**DOI:** 10.1186/s11671-019-2894-1

**Published:** 2019-02-20

**Authors:** Anna Grebinyk, Svitlana Prylutska, Sergii Grebinyk, Yuriy Prylutskyy, Uwe Ritter, Olga Matyshevska, Thomas Dandekar, Marcus Frohme

**Affiliations:** 1 Division Molecular Biotechnology and Functional Genomics, Technical University of Applied Sciences Wildau, Hochschulring 1, 15745 Wildau, Germany; 2 Taras Shevchenko National University of Kyiv, Volodymyrska 64, Kyiv, 01601 Ukraine; 3 Department of Bioinformatics, Biocenter, University of Würzburg, Am Hubland, 97074 Würzburg, Germany; 4 Institute of Chemistry and Biotechnology, University of Technology Ilmenau, Weimarer Straße 25 (Curiebau), 98693 Ilmenau, Germany

**Keywords:** C_60_ fullerene, Doxorubicin, Noncovalent complex, Leukemic cells, Cytotoxicity, Accumulation

## Abstract

Conventional anticancer chemotherapy is limited because of severe side effects as well as a quickly evolving multidrug resistance of the tumor cells. To address this problem, we have explored a C_60_ fullerene-based nanosized system as a carrier for anticancer drugs for an optimized drug delivery to leukemic cells.

Here, we studied the physicochemical properties and anticancer activity of C_60_ fullerene noncovalent complexes with the commonly used anticancer drug doxorubicin. C_60_-Doxorubicin complexes in a ratio 1:1 and 2:1 were characterized with UV/Vis spectrometry, dynamic light scattering, and high-performance liquid chromatography-tandem mass spectrometry (HPLC-MS/MS). The obtained analytical data indicated that the 140-nm complexes were stable and could be used for biological applications. In leukemic cell lines (CCRF-CEM, Jurkat, THP1 and Molt-16), the nanocomplexes revealed ≤ 3.5 higher cytotoxic potential in comparison with the free drug in a range of nanomolar concentrations. Also, the intracellular drug’s level evidenced C_60_ fullerene considerable nanocarrier function.

The results of this study indicated that C_60_ fullerene-based delivery nanocomplexes had a potential value for optimization of doxorubicin efficiency against leukemic cells.

## Introduction

The main efforts in cancer research aim at finding more powerful and selective ways for direct elimination of cancer cells. This task can be addressed with means of nanobiotechnology. Recent progress in this field has arisen interest in a carbon nanostructure — C_60_ fullerene [1] that not only exhibits unique physicochemical properties [2, 3], biological activity [4–10] and antioxidant behavior [11–14], but also possesses a significant potential to serve as a nanocarrier for drug delivery into cancer cells [15–25] (here consistently abbreviated as “C_60_”).

The anticancer anthracycline chemotherapeutic drug Doxorubicin (here abbreviated consistently as “Dox”) is one of the first candidates for a more targeted nanodelivery due to life-threatening cardiotoxicity and other serious side effects [25, 26]. The main mechanism of Dox toxicity against cancer cells is its intercalation into nuclear DNA followed by inhibition of topoisomerase activity, DNA replication, and repair [26–28]. But Dox’s side effects on cardiomyocytes are considered to be determined by another mechanism, mainly, iron-related reactive oxygen species formation [27, 28]. The combination of C_60_ antioxidant potential [2, 11, 13] and its ability for drug delivery [24, 25] makes the nanostructure very attractive for anticancer therapy.

Complexation of Dox with nanostructures increases the drug’s size, both improving its retention in the organism and prolonging the therapeutic activity [29, 30]. To develop an applicable nanosystem for a successful anticancer drug delivery, previous studies focused on aspects regarding stability, biocompatibility, biodistribution and functionality [29–33].

A coupling of Dox and C_60_ for a passive targeting of cancer cells can be achieved by covalent linkage [15–17, 23] or by noncovalent interactions [18–22]. A complex of C_60_ with two amide-linked Dox molecules showed the same cytotoxicity against human breast cancer MCF-7 cells as the free drug [16]. When Dox was bound to C_60_ through a carbamate linker, it exhibited no change in antitumor efficacy but had no systemic toxicity in a murine tumor model [17]. When one or two Dox molecules were anchored on pegylated C_60_ particles through a urethane type bond, the complex exhibited even a delayed antiproliferative effect on MCF-7 cells [23].

For noncovalent complexation of the aromatic Dox molecule with the polyaromatic surface of C_60_, the π-π stacking effect is responsible. In a pioneering attempt, Evstigneev et al. [19] showed a simple and fast method of C_60_ noncovalent complexation with Dox in water [19] and in physiological solution [20]. The proposed nanosystem was shown to have higher toxicity compared with the free drug against various human tumor cell lines in vitro and mice Lewis lung carcinoma in vivo [21, 22]. In another approach, an antimicrobial effect and the applicability for in vivo imaging were shown [18].

The aim of the presented research is to assess the physicochemical properties of the C_60_-Dox complex formed after noncovalent interaction of the components, its intracellular accumulation and сytotoxic potential against human leukemic cells lines.

## Methods/Experimental

### Chemicals

RPMI 1640 liquid medium, phosphate-buffered saline (PBS), fetal bovine serum (FBS), penicillin/streptomycin and L-glutamin were obtained from Biochrom (Berlin, Germany). 3-(4,5-Dimethylthiazol-2-yl)-2,5-diphenyl tetrazolium bromide (MTT) and Hoechst 33342 were obtained from Sigma-Aldrich Co. (St-Louis, USA). Dimethylsulfoxide (DMSO), sodium chloride, acetonitrile, formic acid and trypan blue from Carl Roth GmbH + Co. KG (Karlsruhe, Germany) were used.

### C_60_ and C_60_-Dox Complex Synthesis

The pristine C_60_ aqueous colloid solution was prepared by C_60_ transfer from toluene to water using continuous ultrasound sonication as described by Ritter et al. [34] The obtained C_60_ water colloid solution had a final concentration of 150 μg/ml with 99% purity, stability and homogeneity and an average nanoparticle’s size of 100 nm [34, 35].

Dox (“Doxorubicin-TEVA”, Pharmachemie B.V., Utrecht, Netherlands) was dissolved in physiological solution at an initial concentration of 150 μg/ml.

A C_60_-Dox complex was prepared according to the protocol [20]. Briefly, C_60_ and Dox solutions were mixed in 1:1 or 2:1 weight ratio. The mixture was treated in the ultrasonic disperser for 30 min and stirred magnetically for 24 h at room temperature. The final concentration of both C_60_ and Dox in the C_60_-Dox 1:1 complex was 75 μg/ml. The final concentration of C_60_ and Dox in the C_60_-Dox 2:1 complex was 100 μg/ml and 50 μg/ml, respectively. The unbound drug was washed out with the Pur-A-LyzerTM Midi 1000 Dialysis Kit Sigma-Aldrich Co. (St. Louis, USA). The stability (ζ-potential value) and size distribution (hydrodynamic diameter) [20, 36–39] of complexes were systematically checked and shown to be practically unchanged after 6 months of storage in physiological saline solution. The working concentration of C_60_-Dox complexes in the probes was presented according to Dox-equivalent concentration in the range of 0.1–100 μM purposely to compare the effect of the complexes with the effect of free drug in the same concentration.

### High-Performance Liquid Chromatography-Tandem Mass Spectrometry

Mass spectrometry of the C_60_-Dox complexes after chromatographic separation was achieved with a tandem quadrupole mass spectrometer LCMS-8040, equipped with an electrospray ionization (ESI) source (Shimadzu, Kyoto, Japan) coupled to a Nexera high-performance liquid chromatography (HPLC) system. The latter used an Eclipse XDB-C18 100 mm × 4.6 mm, 3 μM column (Agilent, Santa Clara, USA) with an isocratic mobile phase of acetonitrile and 0.1% formic acid water solution (80:20, *v*/*v*) at a flow rate of 0.3 ml/min. The chromatographic reverse phase conditions and optimized MS/MS parameters are presented in Table 1. For identification and quantification, the molecular ion of Dox was chosen. HPLC-ESI-MS/MS analysis was performed in positive mode by using multiple reaction monitoring (MRM) regime that provides the best sensitivity and accuracy of measurements. After MS/MS-optimization, a unique MRM transition that includes precursor and characteristic product ions was acquired and used for further identification and quantification. The protonated Dox ([M^+^H]^+^, 544.2 m/z) was used as a precursor ion with the most abundant fragment ions of 130.2 and 361.1 m/z.

**Table 1:** HPLC-ESI-MS/MS conditions for analysis of Dox

Chromatographic conditions	
Column	Agilent Eclipse XDB-C18
Column temperature	40 °C
Mobile phase	Acetonitrile, 0.1% formic acid in H_2_O (80:20, *v*:*v*)
Flow rate	0.3 ml/min
Run time	17 min
Injection volume	3 μl
MS/MS conditions	
Ionization source	ESI
• Desolvation line temperature	250 °C
• Heat block temperature	400 °C
Target molecular ion	544.2 [M]^+^ m/z
Product ions	130.2, 361.1 m/z
Time window	0–17 min
Dwell time	0.2 s
Interface voltage	4.5 kV
Nebulizing gas flow	3 l/min
Drying gas flow	15 l/min
LOD	0.005 μM
LOQ	0.015 μM

For data processing, the software LabSolutions HPLC-MS/MS (Shimadzu, Kyoto, Japan) was used. Other parameters were tuned automatically.

Dox calibration standards from 0.005 to 5 μM were prepared from a 1.85 mM water stock solution. The standards were stored in the dark at 4 °C. The calibration curves were plotted with 1/*X* weighting, *r*^2^ = 0.99463. The limits of detection (LOD) and quantification (LOQ) were defined according to LOD = 3.3 × *s*/Slope and LOQ = 10 × *s*/Slope, respectively, where *s* is the standard deviation of the regression line.

### Spectroscopic and Fluorometric Analysis

The absorbance and fluorescence spectra of the free Dox and C_60_-Dox complex were measured at the following parameters: (1) absorbance — wavelength range 400–550 nm, wavelength step size 5 nm, number of flashes per well 25; (2) fluorescence — λ_ex_ = 470 nm, wavelength range 500–800 nm, wavelength step size 2 nm, number of flashes per well 25. A volume of 100 μl of the studied solutions was measured in the 96-well plates Sarstedt (Nümbrecht, Germany) with a multimode microplate spectrometer Tecan Infinite M200 Pro (Männedorf, Switzerland).

### Dynamic Light Scattering

C_60_-Dox complex size distribution was evaluated with a Zetasizer Nano S (Malvern Instruments, UK) equipped with a He-Ne laser (633 nm). Data were recorded at 37 °C in backscattering modus at a scattering angle of 2*θ* = 173°.

### Cell Culture

The human cancer T-cell lines of leucosis origin CCRF-CEM (ACC 240), Jurkat (ACC 282), and Molt-16 (ACC 29) were purchased from the Leibniz Institute DSMZ-German Collection of Microorganisms and Cell Cultures (Deutsche Sammlung von Mikroorganismen und Zellkulturen). The THP1 was kindly provided by Dr. Sofia Cortes (New University of Lisbon, Portugal).

Cells were maintained in RPMI 1640 medium supplemented with 10% fetal bovine serum, 1% penicillin/streptomycin, and 2 mM glutamine, using 25 cm^2^ flasks at a 37 °C with 5% CO_2_ in a humidified incubator Binder (Tuttlingen, Germany). The number of viable cells was counted upon 0.1% trypan blue staining with a Roche Cedex XS Analyzer (Basel, Switzerland).

### Cell Viability

10^4^cells/well were cultured in 96-well cell culture plates Sarstedt (Nümbrecht, Germany) for 24 h. The cell culture medium was replaced by a drug-supplemented medium. Cells were incubated in the presence of varying concentrations of free Dox or C_60_-Dox complex. After 24, 48, and 72 h of incubation, cell viability was determined with the MTT reduction assay [40]. Briefly, 10 μl of MTT solution (5 mg/ml in PBS) was added to each well and cells were incubated for 2 h at 37 °C. The culture medium was then replaced with 100 μl of DMSO, and diformazan formation was determined by measuring absorption at λ = 570 nm with the microplate reader Tecan Infinite M200 Pro (Männedorf, Switzerland). Curve fitting and calculation of the half-maximal inhibitory concentration (IC50) values were done using specialized software GraphPad Prism 7 (GraphPad Software Inc., USA). Briefly, individual concentration-effect curves were generated by fitting the logarithm of the tested compound concentration versus corresponding normalized percent of cell viability values using nonlinear regression.

### Fluorescent Microscopy

CCRF-CEM cells were seeded in 6-well plates Sarstedt (Nümbrecht, Germany) at a cell density of 2 × 10^5^ cells/well in 2 ml of culture medium and incubated for 24 h. Then, cells were treated with 1 μM free Dox or C_60_-Dox complex during 1, 3, and 6 h and washed with PBS. Visualization was performed with a Fluorescence Microscope Keyence BZ-9000 BIOREVO (Osaka, Japan) equipped with red (λ_ex_ = 480 nm, λ_em_ = 600 nm) filter and a respective acquisition software Keyence BZ-II Viewer (Osaka, Japan).

### Flow Cytometry

CCRF-CEM cells (2 × 10^5^/well, 2 ml) were seeded in 6-well plates, incubated for 24 h, and then treated with 1 μM free and C_60_ bound Dox. After 1, 3, and 6 h incubation, the cells were harvested, washed with PBS, and analyzed with the flow cytometer BD FACSJazz™ (Singapore). A minimum of 2 × 10^4^ cells per sample were acquired and analyzed with the BD FACS™ software (Singapore).

### Statistics

All experiments were carried out with a minimum of four replicates. Data analysis was performed with the use of the GraphPad Prism 7 (GraphPad Software Inc., USA). Paired Student’s *t* tests were performed. Difference values *p* < 0.05 were considered to be significant.

## Results and Discussion

### HPLC-MS/MS Analysis of C_60_-Dox Complexes

For chromatographic separation we used the reverse-phase conditions expecting that during the separation process, hydrophobic C_60_ molecules are retained on the column much stronger than those of the more polar Dox [41]. Elution with the polar mobile phase should evidently cause decomposition of the complex and release of free Dox that possesses higher affinity to mobile phase and can be detected by mass spectrometry.

**Fig. 1:**
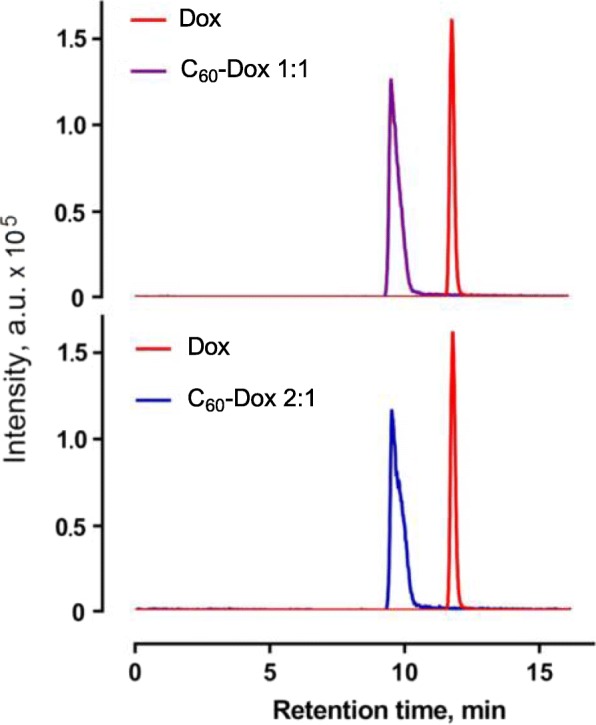
Multiple reaction monitoring chromatograms of free Dox (1 μM), C_60_-Dox 1:1 and C_60_-Dox 2:1 (1 μM Dox-equivalent concentration) complexes under isocratic flow (acetonitrile, 0.1% formic acid in H_2_O, 80:20, *v*:*v*), precursor → product ions transition: 544.2 → 130.2 and 361.1 m/z; a.u. arbitrary units

To confirm the presence of the complex in solution, a concentration of 1 μM Dox was chosen as an optimal for analytical analysis. Under isocratic flow conditions, the retention time for free Dox and Dox as a component of the complexes with C_60_ was different — 11.66 and 9.44 min, respectively (Fig. 1). In addition, the chromatography peaks of Dox released from the complexes were broader and with observed “peak tailing”. Detected shift in retention times as well as different pick shapes indicates that decomposition of C_60_-Dox conjugates on the column fullerene molecules that possess higher affinity to the C18 column. Therefore, nanostructure occupies a part of the active binding sites and interferes Dox’s binding to those sites properly, thereby affecting separation process. That is resulted in shorter retention (reduced time required for Dox to go through the column) as well as peak bordering and tailing for Dox released from the complex as compared to free drug. A very similar phenomenon was observed by Lie et al. [42] during chromatographic separation of C_60_ noncovalent complexes with pullulan. The differences in chromatograms of the free Dox and those released from the complexes evidently pointed out on the presence of C_60_-Dox complexes in solution.

### Spectroscopic and Fluorometric Analysis

**Fig. 2:**
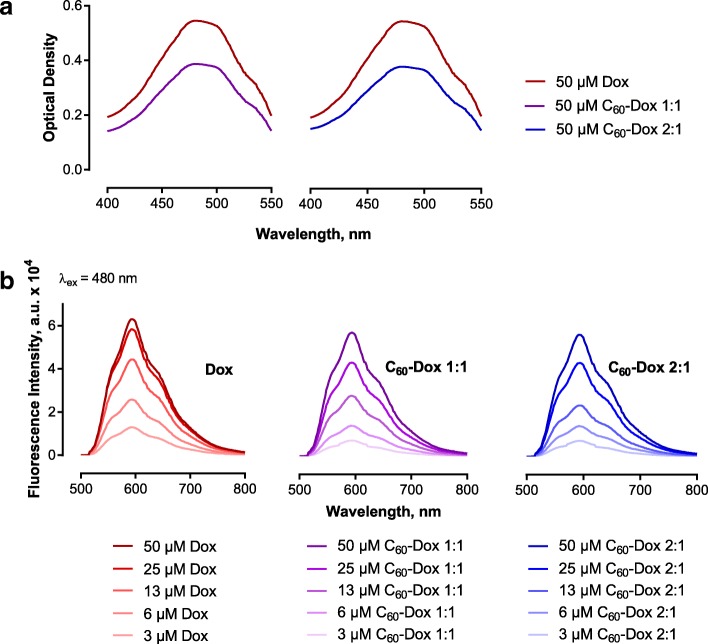
Optical characterization of complexes. Optical density spectra of free Dox and C_60_-Dox complexes (**a**). Fluorescence emission spectra of free Dox and C_60_-Dox complexes at Dox-equivalent concentration from 3 to 50 μM (**b**); a.u. arbitrary units

The optical properties of Dox are determined by electron transition in π-complexed system of its aromatic rings and ketone groups [43]. The typical absorption spectrum of Dox lies in the wavelengths of λ < 600 nm with a broad band at 480 nm (Fig. 2a). The UV/Vis absorption spectrum of pristine C_60_ water colloidal solution has three typical absorption bands with maxima at 220, 265 and 350 nm and a long minor broad tail up to the red region of the visible light [34, 44]. Therefore, the respective control spectra of free C_60_ were subtracted from complex’s spectra. The observed absorption spectra of both 50 μM complexes were similar to those of free 50 μM Dox, but a 30% hypochromic effect was observed (Fig. 2a) indicating a Dox fixation on the C_60_ surface due to π-π stacking interactions.

The long wavelength absorption maximum of Dox (λ = 480 nm) was used as an excitation wavelength for tracking its fluorescence. The fluorescence spectrum exhibits one broad band that consists of three peaks at 560, 594 and 638 nm with a maximum around 594 nm (Fig. 2b) [43], whereas C_60_ has no detectable fluorescence at this spectral band. C_60_-Dox complexes’ fluorescence was estimated in a series of dilutions with Dox-equivalent concentration from 3 to 50 μM. Regardless of dilution, the fluorescence of Dox (λ_ex_ = 480 nm, λ_em_ = 594 nm) in the complexes was quenched by C_60_ moieties (Fig. 2b). Thus, the fluorescence of Dox in both complexes at 3 μM Dox-equivalent concentration appeared to be quenched by 50%. The observed Dox fluorescence quenching is attributed to the strong electron-accepting capability of C_60_ [3] and intramolecular excited-state energy transfer typical for noncovalent Dox complexes [18, 36, 45], indicating on the close spatial proximity of the components.

### Size Distribution Analysis by Dynamic Light Scattering

**Fig. 3:**
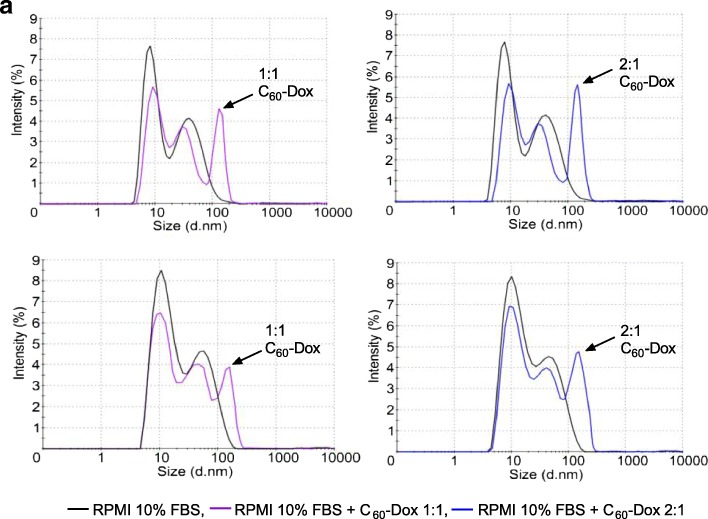
Hydrodynamic size (diameter, nm) of 1 μM С_60_-Dox complexes in RPMI cell culture medium supplemented with 10% FBS at 0 (**a**) and 72-h (**b**) incubation. Intensity (%) percentage of all scattered light intensity

The size and stability of a nanoparticulate anticancer drug is substantially dependent on the cell culture medium composition, ionic strength and protein concentration. The average hydrodynamic diameter of 1 μM C_60_-Dox 1:1 and 2:1 complexes in physiological saline solution (0.9% NaCl) was found to be 135 ± 5 nm and 134 ± 6 nm, respectively, matching the data of previous investigations [20]. To estimate the stability in cell culture medium, 1-μM C_60_-Dox complexes were incubated at 37 °C for 72 h in RPMI supplemented with 10% FBS. The pattern of particle size distribution in this medium (Fig. 3) is attributed to the high protein content as well as its probable aggregation [46, 47].

The dynamic light scattering data on 1 μM C_60_-Dox 1:1 and 2:1 nanocomplex’s hydrodynamic diameter distribution in FBS-supplemented cell culture showed that their size was 138 ± 6 nm and 139 ± 5 nm when measured immediately (Fig. 3a) and 146 ± 4 nm and 144 ± 5 nm after 72 h of incubation (Fig. 3b), respectively.

The detected stability of the maximum (around 140 nm) indicated that there was no additional aggregation of the C_60_-Dox complexes during a prolonged incubation in FBS-supplemented cell culture medium which confirmed their suitability for in vitro studies.

### Cell Viability

Viability of human leukemic cells of different lines was estimated by MTT test at 24, 48, and 72 h of incubation in the presence of C_60_-Dox complexes as well as of free Dox separately at equivalent concentrations. C_60_ alone at concentrations equivalent to those in the complexes had no effect on leukemic cells viability (data not shown).

**Fig. 4:**
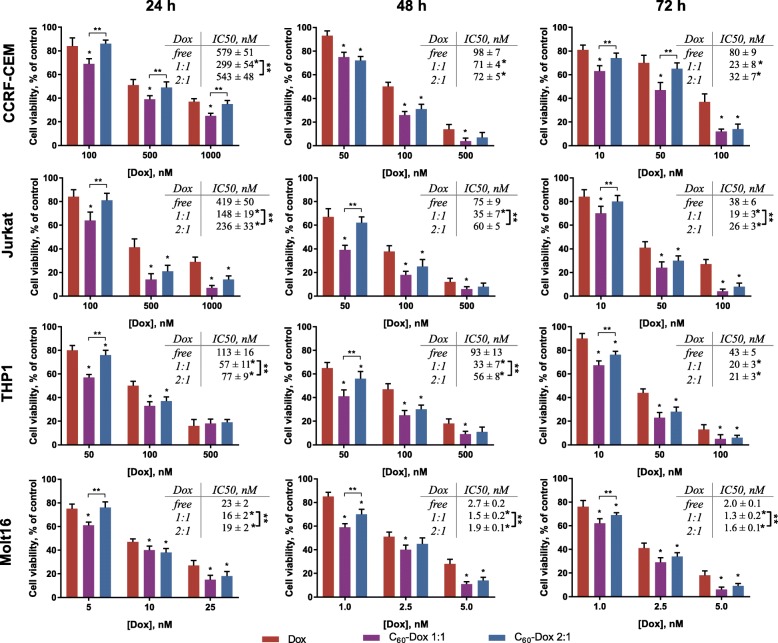
Viability of CCRF-CEM, Jurkat, THP1 and Molt16 leukemic cells, treated with equal doses of free Dox or C_60_-Dox complexes for 24, 48, and 72 h (*p ≤ 0.05 in comparison with the free Dox, **p ≤ 0.05 in comparison with the C_60_-Dox 1:1 complex, *n* = 5)

Figure 4 presents time- and concentration-dependent decrease of leukemic cells viability under Dox treatment. The drug was shown to exhibit toxicity against leukemic cells in the nanomolar range. The sensitivity of leukemic cells to the Dox was found to follow the order Molt-16 ˃ THP1 ˃ Jurkat ˃ CCRF-CEM (less sensitive).

Under action of 100 nM Dox, the viability of CCRF-CEM cells was decreased to 84 ± 7, 50 ± 4 and 34 ± 7% compared to the control at 24, 48 and 72 h, respectively. The comparable pattern of 100 nM Dox toxic effect was found in Jurkat cells. The viability of THP1 cells after treatment with 100 nM Dox cells was found to be 50 ± 4, 47 ± 5, and 13 ± 4% at 24, 48 and 72 h, respectively. Half-maximal inhibitory Dox concentrations (IC50) for CCRF-CEM, THP1 and Jurkat cells at 72 h of incubation were estimated to be 80 ± 9, 43 ± 5 and 38 ± 6 nM, respectively. These data correspond to literature data [48, 49]. Molt-16 cells appeared to be the most sensitive to the drug since its toxic effect was detected in the range from 1 to 25 nM within all periods of cell incubation. The viability of Molt-16 cells treated with 5 nM Dox was decreased to 75 ± 4, 28 ± 4 and 18 ± 4% of that of control at 24, 48 and 72 h, respectively, and the value of IC50 at 72 h was equal to only 2.0 nM. The similar high sensitivity of Molt-16 cells with 10 times more intensive apoptosis induction in comparison with Jurkat cells under treatment of a herbal alkaloid was previously reported by Cai et al. [50].

Cells treated with free Dox were used as a control to assess the viability under action of C_60_-Dox complexes at the equivalent doses of the drug. The value of IC50 for the free Dox and C_60_-Dox complexes was calculated for each time point and cell line and is listed in Fig. 4.

It was shown that both C_60_-Dox complexes possessed higher toxic potential compared to the free Dox against human leukemic cell lines (Fig. 4).

In summary, our numerous experiments showed for the four cell lines a variety of enhanced toxicities up to 3.5-fold. C_60_-Dox 1:1 complex has shown higher toxicity in comparison with 2:1 complex. The less pronounced effect (IC50 decrease on ≥ 2.5 times compared with that for free Dox) of the 2:1 complex can be attributed to the higher concentration of C_60_ as its component. Due to its antioxidant activity [11, 13], excess of C_60_ can protect cells against Dox-associated oxidative stress [27].

### Intracellular Accumulation of Free Dox and C_60_-Dox Complexes

**Fig. 5:**
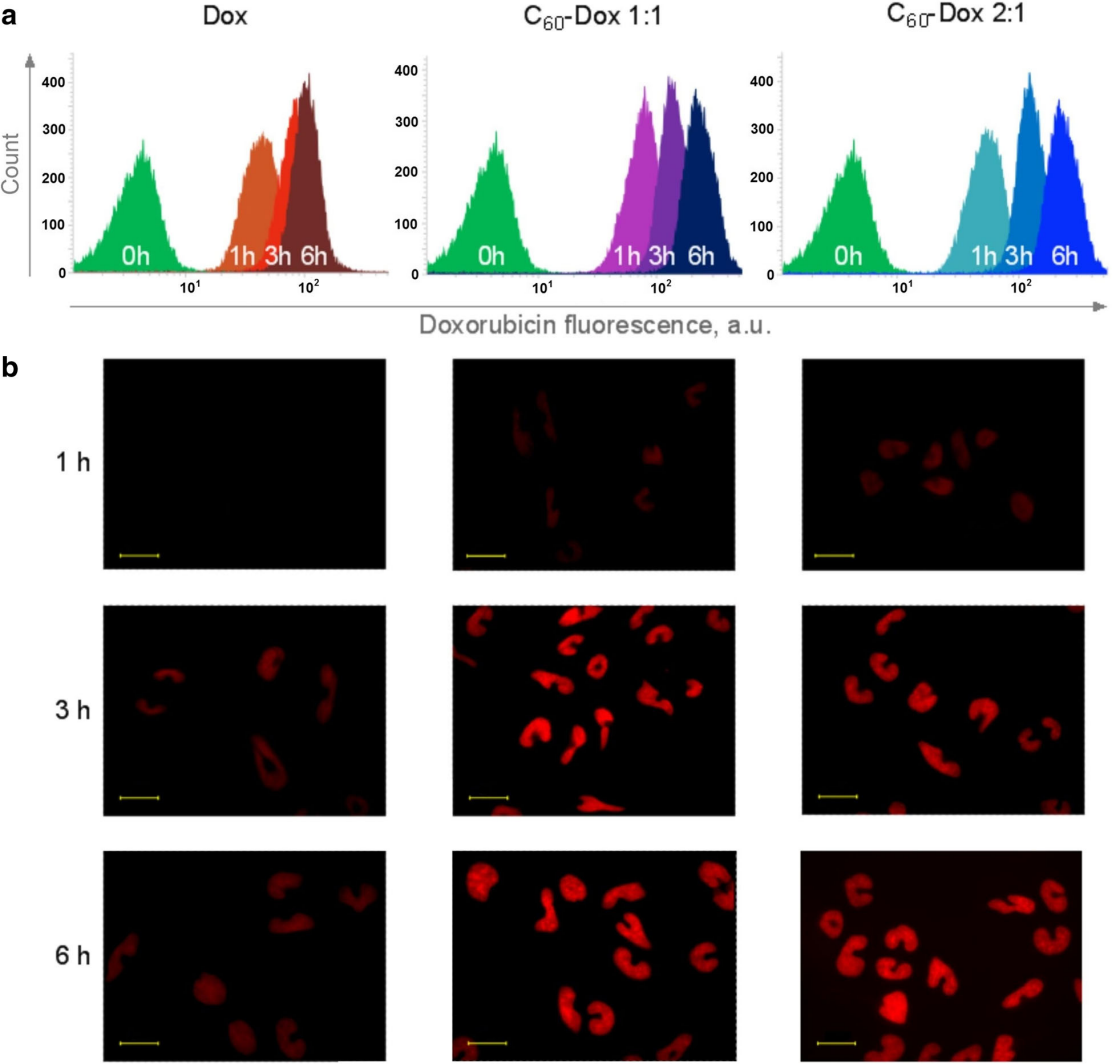
Intracellular accumulation of the 1 μM free and C_60_ complexed Dox. Flow cytometry (**a**) and fluorescent microscopy images (**b**) of CCRF-CEM cells incubated with Dox and C_60_-Dox at the ratio 1:1 and 2:1 for 1, 3 and 6 h. Scale bar 20 μM

To investigate a potential correlation of the enhanced toxic effect of C_60_-Dox complexes with a more effective intracellular drug accumulation, the cellular uptake of free Dox and C_60_-Dox was studied. Since Dox possesses strong absorption and fluorescence in the visible spectral region [43, 45] (Fig. 2), tracking of Dox-complexes is possible with non-invasive direct fluorescent-based techniques. CCRF-CEM cells were incubated in the presence of 1 μM Dox or C_60_-Dox complexes in a drug-equivalent concentration, examined with fluorescent microscopy and subjected to flow cytometry to quantify the intracellular level of accumulated drug after 1-, 3- and 6-h treatment (Fig. 5). The mean fluorescence intensity of each sample was calculated from logarithmic FACS histograms by the value of respective Dox red fluorescent signal (λ_ex_ = 488 nm, λ_em_ = 585/29 nm) and presented in Table 2. Autofluorescence of untreated cells was used as a negative control (Fig. 5a).

**Table 2:** Mean fluorescence intensity (FI) of intracellular accumulated Dox estimated by FACS histograms

FI, a.u.	1 h	3 h	6 h
Dox	45 ± 7	85 ± 9	107 ± 11
1:1 C_60_-Dox	68 ± 9*	145 ± 12*	236 ± 22*
2:1 C_60_-Dox	57 ± 8*	131 ± 21*	234 ± 23*

*p ≤ 0.01 in comparison with the free Dox

Time-dependent accumulation of 1 μM Dox was estimated by fluorescence intensity enhancement (Fig. 5, Table 2). The fluorescence microscopy images illustrate that C_60_-Dox complexes were internalized faster than free drug as evidenced by much brighter intracellular fluorescence (Fig. 5b). The mean fluorescent intensities of the CCRF-CEM cells, treated with 1:1 C_60_-Dox complex at 1 μM Dox-equivalent concentration, were increased in 1.5, 1.7 and 2.2 times compared to free Dox at 1, 3 and 6 h, respectively. 2:1 C_60_-Dox complex exhibited delayed intracellular drug accumulation reaching the same level as 1:1 complex at 6 h (Fig. 5, Table 2).

The obtained data demonstrated that Dox complexation with C_60_ promoted the entry into the cells but did not affect its localization. The control staining of studied cells with DNA binding dye Hoechst 33342 revealed its colocalization with Dox signal (data not shown). Evidently, Dox molecules from C_60_ complexes and the free drug entered the nuclei that reflect its antiproliferative impact through DNA damage [26–28]. An increased drug’s intracellular uptake upon complexation with C_60_ points towards the latter functioning as a drug transport promoter. C_60_ nanostructure was shown to transmigrate the cellular plasma membrane due to passive diffusion [51] and/or endocytosis/pinocytosis [52, 53], whereas such small molecules as Dox can penetrate only via passive diffusion. The C_60_ structure resembles the structure of clathrine [54, 55], the major coat component of vesicle formation during endocytosis. Therefore, C_60_ may function as a transporter of small aromatic molecules [56]. On the contrary, a covalent bond between carrier and cargo introduces a structural alteration into the drug molecule. Consequently, the accumulation pattern and interaction with intracellular targets are altered resulting in complete or partial loss of the drug’s function. Liu et al. [15] showed that C_60_ with two Dox molecules bound through an amide bond was distributed predominantly in the cytoplasm.

## Conclusion

The physicochemical properties of C_60_-Dox complexes with 1:1 and 2:1 ratio of the components were determined, and their toxicity against human leukemic cells CCRF-CEM, Jurkat, Molt-16 and THP1 was estimated.

HPLC-MS/MS analysis revealed evident distinctions in chromatograms of free Dox and those released from C_60_-Dox complexes. Complexation of C_60_ with Dox was confirmed by absorption hypochromic effect and fluorescence quenching in C_60_-Dox complexes. We determined that the size of C_60_-Dox complexes around 140 nm was retained in the presence of protein and prolonged incubation in the medium. Studies on human leukemic cell lines revealed that C_60_-Dox complexes possessed higher cytotoxicity compared to the free drug in equivalent concentrations. At 72 h of incubation of cells, the value of IC50 for 1:1 and 2:1 complexes was decreased on ≤ 3.5 and ≤ 2.5 times, respectively, in comparison with IC50 for the free drug. Complexation with C_60_ promoted Dox entry into leukemic cells. A treatment of CCRF-CEM cells for 6 h with C_60_-Dox complexes in 1 μM Dox-equivalent concentration was followed by 2.2-fold increase of drug intracellular level as compared to treatment with free Dox.

Our results confirm the function of C_60_ as a nanocarrier and the perspective of its application for optimization of Dox efficiency against leukemic cells. As Dox is only a representative or model substance for many antitumor drugs, we expect that our findings may be transferred to other drugs. Increasing a drug’s uptake into tumor cells and/or its antitumor qualities may point towards new treatment strategies. Complexation of drugs with nanocarriers may serve to reduce their efficacious dose rates and thus attenuate the unwanted side effects.

## Abbreviations

C_60_: C_60_ fullerene
DMSO: Dimethylsulfoxide
Dox: Doxorubicin
ESI: Electrospray ionization
FBS: Fetal bovine serum
HPLC-MS/MS: High-performance liquid chromatography-tandem mass spectrometry
IC50: Half-maximal inhibitory concentration
LOD: Limit of detection
LOQ: Limit of quantification
MRM: Multiple reactions monitoring
MTT: 3-(4,5-Dimethylthiazol-2-yl)-2,5-diphenyl tetrazolium bromide
PBS: Phosphate-buffered saline

## References

[1] Kroto HW, Heath JR, O’Brien SC (1985). C_60_: Buckminsterfullerene. Nature.

[2] Delgado JL, Filippone S, Giacalone F (2014). Buckyballs. Top Curr Chem.

[3] Liu T, Troisi A (2013). What makes fullerene acceptors special as electron acceptors in organic solar cells and how to replace them. Adv Mater Weinheim.

[4] Jensen AW, Wilson SR, Schuster DI (1996). Biological applications of fullerenes. Bioorg Med Chem.

[5] Sun C, Wang L, Gao D (2016). C_60_(OH)22: a potential histone deacetylase inhibitor with anti-angiogenic activity. Nanoscale.

[6] Nie X, Tang J, Liu Y (2017). Fullerenol inhibits the cross-talk between bone marrow-derived mesenchymal stem cells and tumor cells by regulating MAPK signaling. Nanomed.

[7] Martinez ZS, Castro E, Seong C-S (2016). Fullerene derivatives strongly inhibit HIV-1 replication by affecting virus maturation without impairing protease activity. Antimicrob Agents Chemother.

[8] Lyon DY, Adams LK, Falkner JC, Alvarez PJJ (2006). Antibacterial activity of fullerene water suspensions: effects of preparation method and particle size. Environ Sci Technol.

[9] Huang L, Bhayana B, Xuan W (2018). Comparison of two functionalized fullerenes for antimicrobial photodynamic inactivation: potentiation by potassium iodide and photochemical mechanisms. J Photochem Photobiol B Biol.

[10] Scharff P, Carta-Abelmann L, Siegmund C (2004). Effect of X-ray and UV irradiation of the C_60_ fullerene aqueous solution on biological samples. Carbon.

[11] Castro E, Hernandez Garcia A, Zavala G, Echegoyen L (2017). Fullerenes in biology and medicine. J Mater Chem B Mater Biol Med.

[12] Scharff P, Ritter U, Matyshevska OP (2008). Therapeutic reactive oxygen generation. Tumori.

[13] Gharbi N, Pressac M, Hadchouel M (2005). [60]fullerene is a powerful antioxidant *in vivo* with no acute or subacute toxicity. Nano Lett.

[14] Piotrovsky LB, Dai L (2006). Chapter 9 - biological activity of pristine fullerene C_60_. Carbon nanotechnology.

[15] Liu J-H, Cao L, Luo PG (2010). Fullerene-conjugated doxorubicin in cells. ACS Appl Mater Interfaces.

[16] Lu F, Haque SA, Yang S-T (2009). Aqueous compatible fullerene-doxorubicin conjugates. J Phys Chem C Nanomater Interfaces.

[17] Chaudhuri P, Paraskar A, Soni S (2009). Fullerenol-cytotoxic conjugates for cancer chemotherapy. ACS Nano.

[18] Blazkova I, Viet Nguyen H, Kominkova M (2014). Fullerene as a transporter for doxorubicin investigated by analytical methods and in vivo imaging. Electrophoresis.

[19] Evstigneev MP, Buchelnikov AS, Voronin DP (2013). Complexation of C_60_ fullerene with aromatic drugs. Chem Phys Chem.

[20] Prylutskyy YI, Evstigneev MP, Cherepanov VV (2015). Structural organization of C_60_ fullerene, doxorubicin, and their complex in physiological solution as promising antitumor agents. J Nanopart Res.

[21] Prylutska SV, Skivka LM, Didenko GV (2015). Complex of C_60_ fullerene with doxorubicin as a promising agent in antitumor therapy. Nanoscale Res Lett.

[22] Panchuk RR, Prylutska SV, Chumakl VV (2015). Application of C_60_ fullerene-doxorubicin complex for tumor cell treatment *in vitro* and *in vivo*. J Biomed Nanotechnol.

[23] Magoulas GE, Bantzi M, Messari D (2015). Synthesis and evaluation of anticancer activity in cells of novel stoichiometric pegylated fullerene-doxorubicin conjugates. Pharm Res.

[24] Montellano A, Da Ros T, Bianco A, Prato M (2011). Fullerene C_60_ as a multifunctional system for drug and gene delivery. Nanoscale.

[25] Kumar M, Raza K (2017). C_60_-fullerenes as drug delivery carriers for anticancer agents: promises and hurdles. Pharm Nanotechnol.

[26] Tacar O, Sriamornsak P, Dass CR (2013). Doxorubicin: an update on anticancer molecular action, toxicity and novel drug delivery systems. J Pharm Pharmacol.

[27] Thorn CF, Oshiro C, Marsh S (2011). Doxorubicin pathways: pharmacodynamics and adverse effects. Pharmacogenet Genomics.

[28] Kizek R, Adam V, Hrabeta J (2012). Anthracyclines and ellipticines as DNA-damaging anticancer drugs: recent advances. Pharmacol Ther.

[29] Fojtu M, Gumulec J, Stracina T (2017). Reduction of doxorubicin-induced cardiotoxicity using nanocarriers: a review. Curr Drug Metab.

[30] Patil RR, Guhagarkar SA, Devarajan PV (2008). Engineered nanocarriers of doxorubicin: a current update. Crit Rev Ther Drug Carrier Syst.

[31] Pillai G (2014) Nanomedicines for cancer therapy: an update of FDA approved and those under various stages of development. SOJ Pharm Pharm Sci 13 10.15226/2374-6866/1/2/00109

[32] Anselmo AC, Mitragotri S (2016). Nanoparticles in the clinic: nanoparticles in the clinic. Bioeng Transl Med.

[33] Schütz CA, Juillerat-Jeanneret L, Mueller H (2013). Therapeutic nanoparticles in clinics and under clinical evaluation. Nanomedicine.

[34] Ritter U, Prylutskyy YI, Evstigneev MP (2015). Structural features of highly stable reproducible C_60_ fullerene aqueous colloid solution probed by various techniques. Fullerenes, Nanotubes Carbon Nanostruct.

[35] Prylutskyy YI, Buchelnikov AS, Voronin DP (2013). C_60_ fullerene aggregation in aqueous solution. Phys Chem Chem Phys.

[36] Prylutskyy YI, Evstigneev MP, Pashkova IS (2014). Characterization of C_60_ fullerene complexation with antibiotic doxorubicin. Phys Chem Chem Phys.

[37] Korolovych VF, Ledin PA, Stryutsky A (2016). Assembly of amphiphilic hyperbranched polymeric ionic liquids in aqueous media at different pH and ionic strength. Macromolecules.

[38] Korolovych VF, Erwin A, Stryutsky A (2018). Thermally responsive hyperbranched poly(ionic liquid)s: assembly and phase transformations. Macromolecules.

[39] Korolovych VF, Cherpak V, Nepal D (2018). Cellulose nanocrystals with different morphologies and chiral properties. Polymer.

[40] Carmichael J, DeGraff WG, Gazdar AF (1987). Evaluation of a tetrazolium-based semiautomated colorimetric assay: assessment of chemosensitivity testing. Cancer Res.

[41] Dorsey JG, Dill KA (1989). The molecular mechanism of retention in reversed-phase liquid chromatography. Chem Rev.

[42] Liu J, Tabata Y (2010). Photodynamic therapy of fullerene modified with pullulan on hepatoma cells. J Drug Target.

[43] Changenet-Barret P, Gustavsson T, Markovitsi D (2013). Unravelling molecular mechanisms in the fluorescence spectra of doxorubicin in aqueous solution by femtosecond fluorescence spectroscopy. Phys Chem Chem Phys.

[44] Grebinyk A, Grebinyk S, Prylutska S (2018). C_60_ fullerene accumulation in human leukemic cells and perspectives of LED-mediated photodynamic therapy. Free Radic Biol Med.

[45] Husseini GA, Kanan S, Al-Sayah M (2016). Investigating the fluorescence quenching of doxorubicin in folic acid solutions and its relation to ligand-targeted nanocarriers. J Nanosci Nanotechnol.

[46] Sabuncu AC, Grubbs J, Qian S (2012). Probing nanoparticle interactions in cell culture media. Colloids Surf B Biointerfaces.

[47] Gollwitzer C, Bartczak D, Goenaga-Infante H (2016). A comparison of techniques for size measurement of nanoparticles in cell culture medium. Anal Methods.

[48] Antunovic M, Kriznik B, Ulukaya E (2015). Cytotoxic activity of novel palladium-based compounds on leukemia cell lines. Anti-Cancer Drugs.

[49] Scott CA, Westmacott D, Broadhurst MJ (1986). 9-alkyl anthracyclines. Absence of cross-resistance to adriamycin in human and murine cell cultures. Br J Cancer.

[50] Cai Z, Lin M, Wuchter C (2001). Apoptotic response to homoharringtonine in human wt p53 leukemic cells is independent of reactive oxygen species generation and implicates Bax translocation, mitochondrial cytochrome c release and caspase activation. Leukemia.

[51] Bedrov D, Smith GD, Davande H, Li L (2008). Passive transport of C_60_ fullerenes through a lipid membrane: a molecular dynamics simulation study. J Phys Chem B.

[52] Russ KA, Elvati P, Parsonage TL (2016). C_60_ fullerene localization and membrane interactions in RAW 264.7 immortalized mouse macrophages. Nanoscale.

[53] Zhang LW, Yang J, Barron AR, Monteiro-Riviere NA (2009). Endocytic mechanisms and toxicity of a functionalized fullerene in human cells. Toxicol Lett.

[54] Schein S (2009). Architecture of clathrin fullerene cages reflects a geometric constraint--the head-to-tail exclusion rule--and a preference for asymmetry. J Mol Biol.

[55] Schein S, Sands-Kidner M (2008). A geometric principle may guide self-assembly of fullerene cages from clathrin triskelia and from carbon atoms. Biophys J.

[56] Borowik A, Prylutskyy Y, Kawelski Ł (2018). Does C_60_ fullerene act as a transporter of small aromatic molecules?. Colloids Surf B Biointerfaces.

