# Correction to: Complexation with C_60_ Fullerene Increases Doxorubicin Efficiency against Leukemic Cells In Vitro

**DOI:** 10.1186/s11671-019-2917-y

**Published:** 2019-03-13

**Authors:** Anna Grebinyk, Svitlana Prylutska, Sergii Grebinyk, Yuriy Prylutskyy, Uwe Ritter, Olga Matyshevska, Thomas Dandekar, Marcus Frohme

**Affiliations:** 1 Division Molecular Biotechnology and Functional Genomics, Technical University of Applied Sciences Wildau, Hochschulring 1, 15745 Wildau, Germany; 2 Taras Shevchenko National University of Kyiv, Volodymyrska 64, Kyiv, 01601 Ukraine; 3 Department of Bioinformatics, Biocenter, University of Würzburg, Am Hubland, 97074 Würzburg, Germany; 4 Institute of Chemistry and Biotechnology, University of Technology Ilmenau, Weimarer Straße 25 (Curiebau), 98693 Ilmenau, Germany

**Correction to: Nanoscale Res Lett**

**https://doi.org/10.1186/s11671-019-2894-1**

Following publication of the original article [1], the authors flagged that there was unfortunately an error with Fig. 3 of the article.

The error was that the preceding figure, Fig. 2, had been duplicated, duplicating in place of the figure (not including the caption) of Fig. 3. That is, Fig. 3 had been replaced by a duplicate of Fig. 2 through an erroneous error in the production of the article.

Please be advised that the original article has now been updated to correct this error.

The publisher apologizes for this processing error.
